# 3-Methyladenine but not antioxidants to overcome BACH2-mediated bortezomib resistance in mantle cell lymphoma

**DOI:** 10.1186/s12935-021-01980-2

**Published:** 2021-05-26

**Authors:** Min Feng, Jia Wang, Ming Sun, Guilan Li, BingXiang Li, Han Zhang

**Affiliations:** 1 Institute of Medical Biology, Chinese Academy of Medical Sciences and Peking Union Medical College, 935 Jiaoling Road, Kunming, 650118 Yunnan China; 2 School of Life Sciences, Yunnan University, Kunming, 650500 Yunnan China

## Abstract

**Background:**

Bortezomib (BTZ) is an inhibitor of the proteasome that has been used to treat patients with mantle cell lymphoma (MCL), but the resistance to BTZ in clinical cases remains a major drawback. BACH2 is a lymphoid-specific transcription repressor recognized as a tumor suppressor in MCL. Reduced BACH2 levels contribute to BTZ resistance; however, the molecular events underlying BACH2-mediated BTZ resistance are largely unclear.

**Methods:**

We silenced BACH2 in MCL cells using a lentiviral shRNA-mediated knockdown system. Bioinformatic, real-time RT-PCR, immunoblotting and a series of functional assays were performed to describe the molecular mechanisms underlying BTZ resistance in MCL. The therapeutic effects of chemicals were evaluated on numerous cellular and molecular processes in resistant MCL cell lines and xenografts.

**Results:**

In resistant cells, BTZ-triggered mild oxidative stress induced a strong activation of PI3K-AKT signaling, which further blocked nuclear translocation of BACH2. Defective nuclear translocation of BACH2 or silencing BACH2 removed its transcriptional repression on *HMOX1*, leading to upregulation of heme oxygenase-1 (HO-1). Increased HO-1 further maintained reactive oxygen species (ROS) within a minimal tumor-promoting level and enhanced cytoprotective autophagy. Interestingly, although mild increase in ROS exhibited a pro-tumorigenic effect on resistant cells, simply blocking ROS by antioxidants did not lead to cell death but aggravated BTZ resistance via stabilizing BACH1, the other member of BACH family. Instead, 3-methyladenine (3-MA), a dual inhibitor to suppress PI3K signaling and autophagosome formation, sensitized resistant MCL cells to BTZ, both in vitro and in vivo.

**Conclusion:**

Our results dissected the interconnected molecular network in resistant MCL cells in which 3-MA represents an effective therapeutic strategy to overcome BTZ resistance. Notably, BACH1 and BACH2, albeit from the same family, are likely to play opposite roles in pathogenesis and progression of MCL.

## Background

Mantle cell lymphoma (MCL) is a rare and aggressive subtype of B-cell lymphoma, accounting for about 6% of non-Hodgkin’s lymphoma (NHL). MCL generally affects older individuals with a median age at diagnosis of around 70 years [1, 2]. Despite improved therapeutic responses with novel agents [3–5], MCL patients often relapse with short survival and remain having one of the worst clinical outcomes of all the lymphomas [6, 7]. Thus, therapeutic improvement in MCL not only depends on developing novel targeted agents, but also relies on identifying more effective approaches to overcome chemoresistance.

Bortezomib (BTZ) is a reversible inhibitor of the proteasome, which has been approved by the United States Food and Drug Administration (FDA) as a single-agent treatment for patients with relapsed/refractory MCL [8–10]. Although BTZ shows promising activity in MCL patients [4, 8–10], disease relapse following BTZ therapy is frequent. As such, BTZ resistance remains a major limitation for its therapeutic use in clinics [11].

BTZ elicits cytotoxicity against MCL cells by triggering excessive reactive oxygen species (ROS) [12]. Interestingly, a prominent activation of oxidative stress pathways has been found in BTZ-sensitive MCL cells, whereas BTZ-resistant MCL cells exhibit only a minimal increase in ROS with high basal antioxidant capacity, indicating selective anti-tumor activity of BTZ in MCL cells [12]. Further study identified that the subcellular localization of BTB and CNC homology 2 (BACH2), a lymphoid-specific transcription repressor recognized as a tumor suppressor in MCL [13], determines oxidative stress responses to BTZ in MCL. BTZ-induced ROS stimulates nuclear import of BACH2 to suppress antioxidant and anti-survival genes in BTZ-sensitive MCL cells, leading to cell death, whereas in resistant cells, BACH2 cannot translocate to the nucleus [14]. In addition, BTZ-induced ROS triggers cytoprotective autophagy in resistant cells [15], and silencing BACH2 contributes to BTZ resistance [13]. However, none of these studies delineate what causes defective nuclear translocation of BACH2 in BTZ-resistant MCL cells, and which pathways are involved in BTZ resistance upon BACH2 silencing. Nor is clear whether there is a correlation between defective BACH2 nuclear translocation and autophagy alterations in resistant MCL cells. Given many missing links, it remains ill-defined what molecular events determine BTZ-induced cytotoxicity, and more importantly, can we find a better way to overcome BTZ resistance in MCL? Thus, we sought to integrate multiple lines of evidence together and find the crosslink between each other.

In the present study, we found that a minimal increase of ROS in BTZ-resistant MCL cells greatly activated PI3K-AKT pathway, which further caused defective nuclear translocation of BACH2, thus removing its transcriptional repression on heme oxygenase-1 (*HMOX1*). Upregulated HO-1, encoded by the *HMOX1*, further maintained ROS within a tumor-promoting level and induced cytoprotective autophagy formation. This network facilitates an effective therapy with 3-methyladenine (3-MA) to overcome BTZ resistance by dual inhibition of PI3K-AKT signaling and autophagy formation. Unexpectedly, we identified a new potential resistant mechanism by which the antioxidant supplements, in spite of lowing tumor-promoting ROS levels, lead to another cytoprotective effect in BTZ-resistant MCL cells.

## Methods

### Cell lines and culture

Human MCL cell lines Jeko and REC-1 were purchased from BNCC (Beijing, China). Cell lines were authenticated using short tandem repeats at CinoAsia Institute (Shanghai, China). Cells were maintained under 5% CO_2_ at 37 °C and cultured in RPMI1640 medium supplemented with 10% FBS and 100 U.I./ml penicillin–streptomycin.

### Lentivirus generation and infection

293 T cells were transfected with either lentiviral shRNAs specific for human BACH2 (GE Dharmacon, clone ID: V3LHS_409004, Pittsburgh, PA, USA), or a non-silencing lentiviral shRNA control plasmid (GE Dharmacon). Lentiviruses were collected 48 h post-transfection. Cells were then infected with lentiviruses using polybrene (8 μg/ml). Lentiviral-transduced cells were selected with puromycin (2 μg/ml) for 14 days.

### Chemicals and antibodies

BTZ, *N*-acetylcysteine (NAC), 3-MA and chloroquine (CQ) were purchased from Sigma-Aldrich (St Louis, MO, USA). Tin protoporphyrin IX dichloride (SnPP) was purchased from Santa Cruz Biotechnology (Dallas, TX, USA). Cycloheximide (CHX) was purchased from Beyotime Biotechnology (Shanghai, China). Briefly, BTZ, SnPP and CHX were dissolved in DMSO, respectively; NAC, 3-MA and CQ were dissolved in double distilled water, respectively. For in vivo injection, the BTZ stocks were further diluted in a pyrogen-free sterile 0.9% NaCl solution, and 3-MA was freshly prepared in a pyrogen-free sterile 0.9% NaCl solution.

Anti-human-CD45-FITC was purchased from BD Biosciences (San Jose, CA, USA) for flow cytometry (FCM) analysis. The following antibodies were used for immunoblots: anti-BACH2, anti-AKT, anti-phospho-AKT, anti-HO-1, anti-NRF2, anti-GAPDH, anti-β-Actin and anti-TBP (Cell Signaling, Danvers, MA, USA); anti-LC3 (Novus Biologicals, Littleton, CO, USA) and anti-BACH1 (Santa Cruz Biotechnology, Dallas, TX, USA).

### Immunoblotting assay and semi-quantitative analysis

Harvested cells were lysed to perform immunoblotting assay as previously described [16]. Immunoblotting was subjected to semi-quantitative analysis using an ImageJ software.

### Subcellular distribution analysis

Cytoplasmic and nuclear fractions of MCL cells were isolated using a Nuclear Extract Kit (Active Motif, Carlsbad, CA, USA) according to the manufacturer’s instruction. The fractions were used for immunoblotting assay with β-Actin and TATA-binding protein (TBP) as cytoplasmic and nuclear loading controls, respectively.

### Cell viability assay

MCL cells were treated with BTZ alone or combined with NAC or 3-MA or CQ for 24 h, and cytotoxicity was assessed with fluorometric method using CellTiter-Blue^®^ (Promega, Madison, WI, USA), as previously described [16].

### ROS assessment

Intracellular ROS in MCL cells was assessed by employing a specific cell permeable fluorescent probe 2′, 7′-dichlorofluorescein diacetates (Sigma-Aldrich, St Louis, MO, USA), as previously described [16]. Results were relative to the mean fluorescence intensity (MFI) of the control cells.

### RNA isolation and real-time RT-PCR

RNA was isolated as previously described [13], followed by real-time RT-PCR using a One Step SYBR PrimeScript PLUS RT-PCR kit (Takara, Kusatsu, Japan). The relative expression of gene was normalized to the *ACTB* by the method of 2^−ΔΔCt^. The involved primers are shown as follows: *BACH1*: 5′-CTCAGCCTTAATGACCAGCGG-3′, 5′-GCCTACGATTCTTGAGTGGAAG-3′; *ACTB*: 5′-CATGTACGTTGCTATCCAGGC-3′, 5′-CTCCTTAATGTCACGCACGAT-3′.

### Microarray data analysis

Microarray data from 100 patients with lymphomas including diffuse large B cell lymphoma (DLBCL, n = 60), Burkitt lymphoma (BL, n = 17), follicular lymphoma (FL, n = 6), MCL (n = 8), and primary effusion lymphoma (PEL, n = 9) [17] as well as microarray data from 57 patients with MCL [18] were downloaded from the GEO database (http://www.ncbi.nlm.nih.gov/geo/; GSE2350 and GSE21452, respectively). The log2 of mRNA expression values for *BACH1* and *CCND1* were used for analysis.

### Drug combination assay

The synergic cytotoxic effects of BTZ and 3-MA were determined by combination index (CI) method as previously described [19]. CI plots were generated using a CompuSyn software. Briefly, synergy is present when the CI is less than 1.0, additive effect is when CI equals 1.0, and antagonism is when CI greater than 1.0.

### Tumor xenografts and chemotherapy treatment

Non-obese diabetic/severe combined immunodeficient (NOD/SCID) mice (5 weeks old) were purchased from Charles River Laboratories (Beijing, China), and were housed in the barrier conditions at Institute of Medical Biology (IMB). All animal procedures were approved by the IMB Animal Care Committee. Mice were pre-treated with an intraperitoneal (i.p.) injection of CTX at a dose of 100 mg/kg once daily for two consecutive days. REC-1 cells (5 × 10^6^ cells/mouse) were then injected subcutaneously (s.c.), and tumor growth was monitored weekly. The treatment was commenced when the average tumor volume reached 50 mm^3^. Xenografts were then divided into two groups (n = 3/group), where xenografts #1–#3 were treated with BTZ alone and xenografts #4–#6 were treated with BTZ combined with 3-MA. BTZ was administered at a dose of 0.5 mg/kg once a week (day 1) [20], and 3-MA was administered at a dose of 15 mg/kg once a week (day 2). No drugs were administered on days 3–7. Treatment was continued for two consecutive weeks (days 1–14) and xenografts were humanely sacrificed by cervical dislocation on day 15. The s.c. tumors were isolated for immunoblots; spleen (SP) and bone marrow (BM) cells were isolated to analyze MCL infiltration by determining the percentage of positive human CD45 (hCD45^+^) cells using FCM as previously described [13]. The volumes of tumors were measured by the formula: tumor volume = 1/2 (length × width^2^), where length is the longest longitudinal diameter and width is the greatest transverse diameter [21].

### Statistical analysis

Data reported are described as experimental mean ± standard error of mean (SEM) or standard deviation (SD). Statistical significance of differences between control and experimental groups was evaluated by the Student *t* test, where *p < 0.05, **p < 0.01 and ***p < 0.001 are considered statistically significant. All experiments and assays were repeated at least twice and performed in duplicate or triplicate.

## Results

### Mild oxidative stress mediates defective nuclear translocation of BACH2 by a strong activation of PI3K-AKT pathway in BTZ-resistant cells

**Fig. 1:**
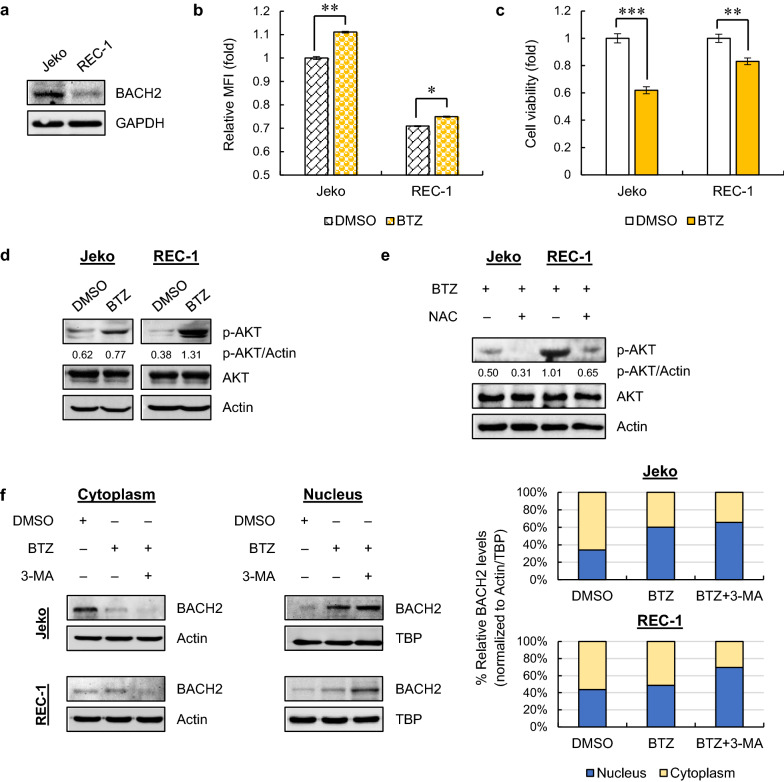
Mild ROS production leads to defective nuclear translocation of BACH1 via activating PI3K-AKT pathway in resistant MCL cells. **a** Immunoblots for BACH2 in Jeko and REC-1 cells with GAPDH as a loading control. **b** ROS production was measured in cells treated with DMSO (control) or BTZ (20 nM) for 24 h. Relative MFI values compared to control are shown as the mean ± SD from two independent experiments. **c** Cells were treated with DMSO or BTZ (20 nM) for 24 h. Cell viability was determined using MTT assays. Data are normalized to control and shown as the mean ± SD from three independent experiments. *p < 0.05; **p < 0.01; ***p < 0.001 (vs DMSO-treated group). **d** Immunoblots for p-AKT and AKT in cells with DMSO or BTZ treatment (20 nM), with Actin as a loading control. The indicated values of p-AKT under each lane are normalized to Actin. **e** Cells were treated with BTZ (20 nM) with or without 1 h-pretreatment of antioxidant NAC (100 μM) for 24 h. p-AKT and AKT were measured using immunoblotting with Actin as a loading control. The normalized values (p-AKT/Actin) in each lane are indicated. **f** Immunoblots for BACH2 in the cytoplasmic and nuclear fractions of MCL cells treated with BTZ in the presence or absence of 3-MA (5 mM) for 24 h (left). Cells treated with DMSO were used as negative controls. Actin and TBP were used as loading controls for cytoplasmic and nuclear proteins, respectively. The % proportion of the relative BACH2 levels in cytoplasm and nucleus are indicated (right)

Since BACH2 plays a tumor-suppressor role in MCL and decreased BACH2 levels lead to BTZ resistance [13], we first determined the BACH2 levels in one BTZ-sensitive cell line Jeko and one BTZ-resistant MCL cell line REC-1 [12]. Compared to Jeko cells, REC-1 cells showed remarkably lower BACH2 levels (Fig. 1a) and relatively low baseline ROS production (Fig. 1b). After 24 h of BTZ treatment, only mild increase in ROS and little cell death were detected in REC-1 cells compared to those in Jeko cells (Fig. 1b, c), in agreement with what was reported [12].

Earlier studies have shown that BACH2 can be phosphorylated by PI3K in leukemic cells, allowing BACH2 to retain in the cytoplasm [22]. In MCL, BACH2 fails to migrate to the nucleus in response to BTZ in resistant cells [14]. However, it remains unclear whether PI3K pathway is activated upon BTZ treatment in MCL, and whether defective nuclear translocation of BACH2 in resistant cells is caused by PI3K activation. To answer these questions, we first showed dramatically higher phosphorylated AKT (p-AKT) levels in REC-1 cells than those in Jeko cells upon BTZ treatment (Fig. 1d), suggesting a strong activation of PI3K-AKT pathway in BTZ-resistant cells. Further pretreatment with antioxidant NAC let to a significant decrease of p-AKT levels (Fig. 1e), indicating that PI3K-AKT signaling is activated by the BTZ-induced ROS. Next, experiments were performed to determine the subcellular distribution of BACH2 in MCL cells after BTZ treatment. In sensitive Jeko cells, the cytoplasmic fractions of BACH2 were significantly decreased upon BTZ treatment, whereas higher localization of BACH2 in the nuclear was observed. In contrast, BACH2 in resistant REC-1 cells was mainly localized in the cytoplasm, while only a few amounts of BACH2 proteins were translocated in the nuclear after BTZ treatment, indicating a defective nuclear translocation of BACH2 (Fig. 1f). After adding PI3K inhibitor 3-MA, the nuclear translocation of BACH2 was significantly enhanced in REC-1 cells compared to that after BTZ single treatment (Fig. 1f), demonstrating that the defective nuclear translocation of BACH2 in resistant cells is mediated by activation of PI3K-AKT pathway.

### BACH2 silencing leads to upregulation of HO-1 and confers BTZ resistant properties to MCL via activating PI3K-AKT and autophagy pathways

**Fig. 2:**
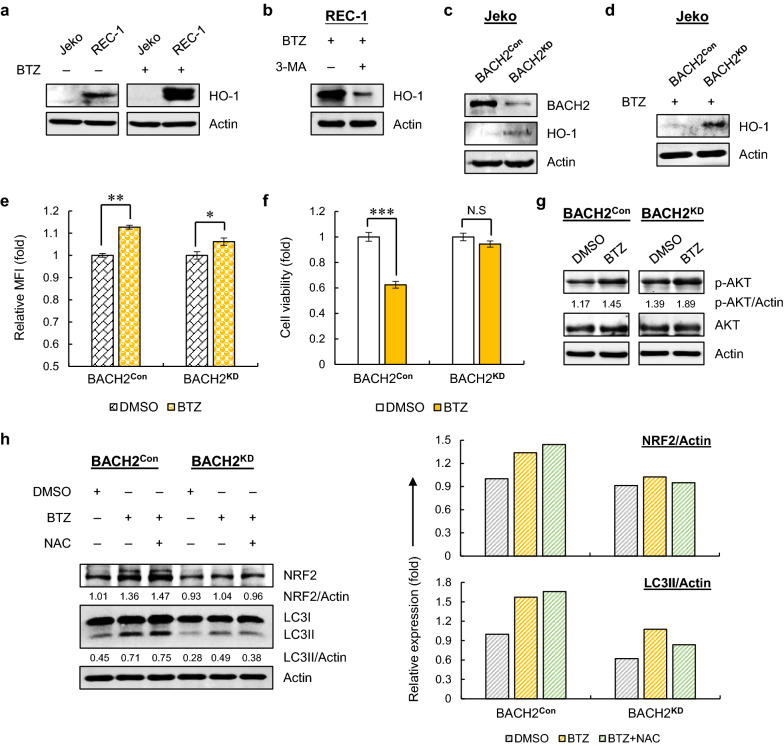
BACH2 blockade leads to upregulation of HO-1 and confers BTZ-resistant properties to MCL. **a** Immunoblots for HO-1 in cells with or without BTZ treatment (20 nM) for 24 h. Actin was used as a loading control. **b** HO-1 was measured using immunoblotting in REC-1 cells treated with BTZ (20 nM) in the presence or absence of 3-MA (5 mM) for 24 h, with Actin as a loading control. **c** The knockdown efficiency of BACH2 (BACH2^KD^) in Jeko cells was evaluated with a non-silencing shRNA plasmid (BACH2^Con^) as a negative control. The basal levels of HO-1 were measured in BACH2^KD^ and BACH2^Con^ cells with Actin as a loading control. **d** Immunoblots for HO-1 after BTZ treatment (20 nM). Actin was used as a loading control. **e** ROS levels in manipulated Jeko cells with DMSO or BTZ treatment (20 nM) for 24 h. Relative MFI values are shown as the mean ± SD from two independent experiments. **f** Cell viability was measured in cells treated with DMSO or BTZ (20 nM) for 24 h. Data are normalized to control and shown as the mean ± SD from three independent experiments. N.S, not significant; *p < 0.05; **p < 0.01; ***p < 0.001 (vs DMSO-treated group). **g** Immunoblots for p-AKT and AKT in BACH2^KD^ and BACH2^Con^ cells treated with DMSO or BTZ (20 nM). The normalized values (p-AKT/Actin) in each lane are indicated. **h** NRF2 and LC3II were measured in manipulated Jeko cells treated with BTZ (20 nM) with or without NAC pretreatment (100 μM) for 24 h (left). The relative expressions of NRF2 (NRF2/Actin) and LC3II (LC3II/Actin) in BACH2^KD^ and BACH2^Con^ are indicated, respectively (right). Data are normalized to DMSO-treated BACH2^Con^ cells

Excessive ROS is highly toxic to cells and cause cell damage. To guard against the toxicity, ROS levels are tightly controlled by an antioxidant system in the human body [23]. HO-1 is one of the key protective mechanisms to defend against elevated ROS levels. Interestingly, *HMOX1*, which encodes HO-1, is a direct downstream target of BACH2 and is repressed by BACH2 [24]. To investigate whether HO-1 is involved in BACH2-mediated BTZ resistance in MCL, we first detected the expression levels of HO-1 in Jeko and REC-1 cells. As shown in Fig. 2a, REC-1 cells displayed higher basal levels of HO-1 compared with Jeko cells, coinciding with its lower BACH2 levels (Fig. 1a), and such differences became more significant after BTZ treatment. Notably, inducible levels of HO-1 in REC-1 cells can be reduced by PI3K inhibitors (Fig. 2b), suggesting that the remarkable BTZ-induced upregulation of HO-1 in REC-1 cells may be attributed to defective nuclear translocation of BACH2, thus removing the transcriptional repression of BACH2 on *HMOX1*.

To further delineate the effect of BACH2 on HO-1, we silenced BACH2 (BACH2^KD^) in Jeko cells using a lentiviral shRNA-mediated knockdown system. Silencing BACH2 led to upregulation of both basal and inducible levels of HO-1 compared to control cells (Fig. 2c, d). Correspondingly, exposure of BACH2^KD^ cells to BTZ caused a mild increase in ROS and little cytotoxicity (Fig. 2e, f), very similar responses as shown in REC-1 cells (Fig. 1b, c), exhibiting a BTZ-resistant property of BACH2^KD^ cells. This finding suggested that higher intracellular HO-1 levels in BACH2^KD^ cells may lead to reduced responsiveness of BACH2^KD^ cells to BTZ by maintaining ROS at a relatively low level. In addition, silencing BACH2 also caused a strong activation of PI3K-AKT pathway upon BTZ treatment compared to control cells (Fig. 2g).

Given that BTZ-induced ROS could trigger cytoprotective autophagy formation in BTZ-resistant cells [15], we then questioned whether BACH2 blockade induces autophagy after BTZ treatment. In BACH2^KD^ cells, BTZ single treatment enhanced autophagy, which could be attenuated by pretreatment of NAC, suggesting that BTZ-induced ROS triggers autophagy in resistant BACH2^KD^ cells (Fig. 2h). In contrast to high BTZ-induced HO-1 levels (Fig. 2d), only a minimal increase in NRF2, another essential antioxidant factor, was induced by BTZ in BACH2^KD^ cells, whereas a strong upregulation of NRF2 was triggered in control cells (Fig. 2h). This finding is supported by a previous study demonstrating that sensitive MCL cells display a prominent NRF2 upregulation in response to BTZ but the resistant MCL cells do not [12]. Of particular interest, pretreatment of NAC led to even higher levels of NRF2 and autophagy in control cells (Fig. 2h), implying that distinct pathways are likely to be induced when blocking ROS in sensitive cells.

### Upregulation of HO-1 detoxifies BTZ-induced ROS and enhances cytoprotective autophagy to facilitate chemoresistance

**Fig. 3:**
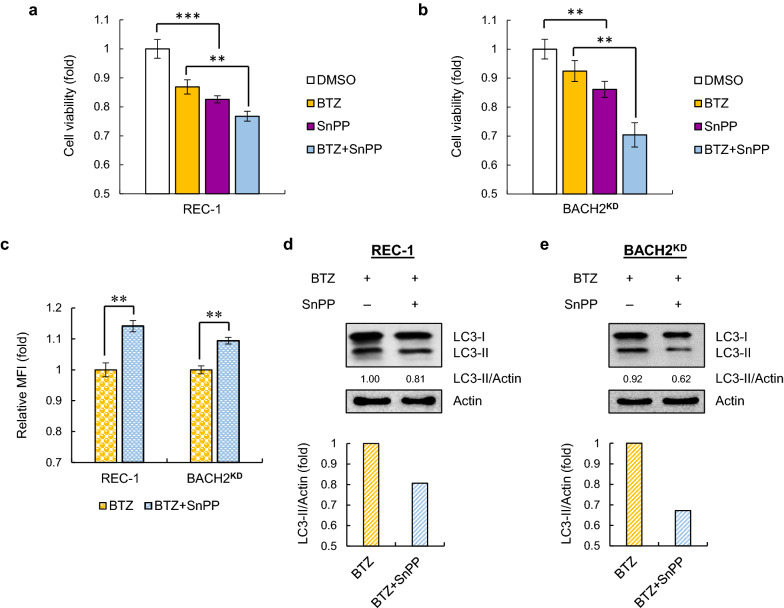
Blocking HO-1 sensitizes resistant MCL cells to BTZ by enhancing cytotoxic ROS levels and reducing cytoprotective autophagy formation. REC-1 (**a**) and BACH2^KD^ Jeko cells (**b**) were treated with BTZ (20 nM) with or without 1 h-pretreatment of SnPP (5 μM) for 24 h. Cells treated with DMSO or SnPP were used as negative controls. Cell viability was measured using MTT assays. Data are normalized to DMSO-treated cells and shown as the mean ± SD from three independent experiments. **p < 0.01; ***p < 0.001 (vs DMSO-treated group or single BTZ-treated group). **c** ROS assessment in resistant cells treated with BTZ (20 nM) in the presence or absence of SnPP (5 μM) for 24 h. Data are normalized to single BTZ-treated cells and shown as the mean ± SD from two independent experiments. **p < 0.01 (vs single BTZ-treated group). LC3II levels were measured using immunoblotting in REC-1 (**d**) and BACH2^KD^ Jeko cells (**e**) treated with BTZ (20 nM) with or without SnPP pretreatment (5 μM) for 24 h, respectively. Actin was used as a loading control, and the normalized values (LC3II/Actin) are indicated

HO-1 has been reported to mediate chemoresistance by promoting autophagy in multiple cancers [25, 26]. To gain more insight into the relationship among HO-1, ROS and autophagy, we pretreated BTZ-resistant cells with specific HO-1 inhibitor SnPP. As shown in Fig. 3a, b, blocking HO-1 activity significantly led to higher sensitivity of resistant REC-1 and BACH2^KD^ Jeko cells to BTZ. The cytotoxic ROS levels were also increased after combination of BTZ with SnPP in resistant cells (Fig. 3c), suggesting that the mild ROS production upon BTZ treatment in resistant cells is caused by high inducible levels of HO-1.

In addition, pretreatment of SnPP led to a reduced autophagy formation in both REC-1 and BACH2^KD^ Jeko cells (Fig. 3d, e), suggesting that the enhanced cytoprotective autophagy caused by decreased BACH2 is partly mediated by upregulation of HO-1 in BTZ-resistant MCL cells.

### Antioxidant treatment fails to overcome BTZ resistance via stabilization of BACH1 in resistant MCL cells

**Fig. 4:**
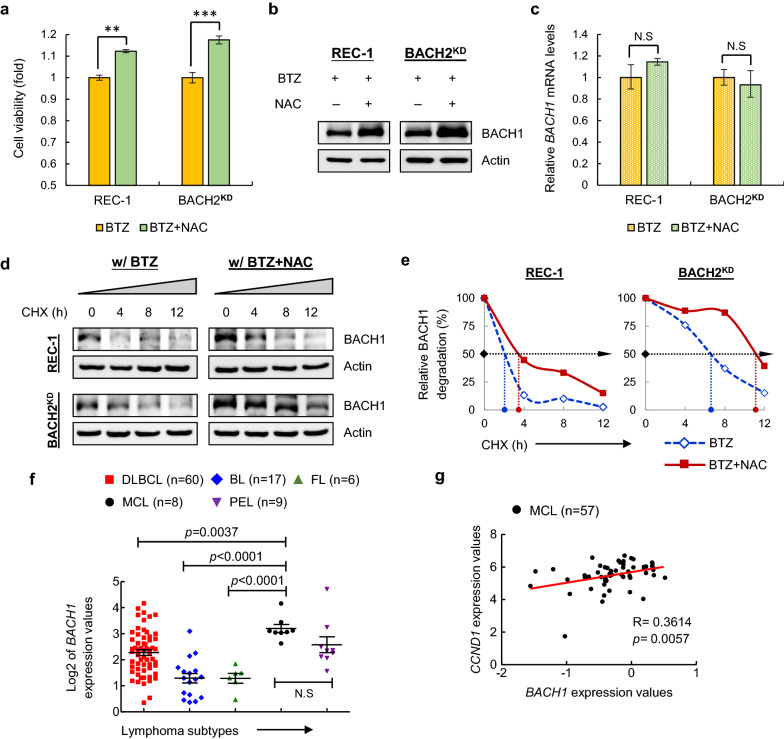
Antioxidant NAC aggravates chemoresistance via stabilizing BACH1 in BTZ-resistant MCL cells. **a** Cell viability was measured in resistant cells treated with BTZ (20 nM) with or without NAC pretreatment (100 μM) for 24 h. Data are normalized to single BTZ-treated cells and shown as the mean ± SD from three independent experiments. **p < 0.01; ***p < 0.001 (vs single BTZ-treated group). BACH1 protein levels (**b**) and *BACH1* mRNA levels (**c**) in REC-1 and BACH2^KD^ Jeko cells treated with BTZ (20 nM) with or without NAC pretreatment (100 μM) for 24 h. Actin was used as a loading control for immunoblotting. Each value from real-time RT-PCR was normalized to *ACTB* and is presented as the mean ± SD from three independent experiments. N.S, not significant (vs single BTZ-treated group). **d** REC-1 and BACH2^KD^ Jeko cells were treated with BTZ (20 nM) in the presence or absence of NAC (100 μM) for 16 h, followed by addition of CHX (50 μg/mL) at the indicated time. BACH1 proteins were measured using immunoblotting with Actin as a loading control. **e** The relative half-life (t_1/2_) of BACH1 (50% of degradation, black arrow) is indicated in the diagram. Blue dotted lines represent t_**1/2**_ of BACH1 in REC-1 and BACH2^KD^ Jeko cells after single-BTZ treatment, whereas red dotted lines represent t_**1/2**_ of BACH1 in REC-1 and BACH2^KD^ Jeko cells treated with BTZ and NAC. **f** *BACH1* mRNA levels in patients with MCL (n = 8) compared to patients with other subtypes including diffuse large B cell lymphoma (DLBCL, n = 60), Burkitt lymphoma (BL, n = 17), follicular lymphoma (FL, n = 6) and primary effusion lymphoma (PEL, n = 9). Data are shown as the mean ± SEM. N.S, not significant. **g** Correlation analyses of *BACH1* and *CCND1* expression values based on microarray data in patients with MCL (n = 57). *R* value and *p* value are indicated

Since mild increase in ROS in BTZ-resistant MCL cells induces strong activation of PI3K signaling and cytoprotective autophagy, we next questioned whether antioxidants could overcome BTZ resistance via blocking ROS. Unexpectedly, pretreatment with antioxidant NAC did not increase BTZ-induced cell death, but acted in a pro-tumorigenic fashion in both REC-1 and BACH2^KD^ Jeko cells (Fig. 4a). Although NAC could reduce ROS in resistant cells, antioxidant supplements might promote tumor progression and chemoresistance by stimulating other pathways.

Interestingly, recent studies have provided compelling evidence that antioxidants accelerate progression and metastasis of lung cancer by stabilization of the transcription factor BTB and CNC homology 1 (BACH1) [27, 28], which has also been identified as a master regulator of breast cancer bone metastasis [29]. Of note, BACH1, along with BACH2, constitutes a subfamily of the basic region-leucine zipper (bZIP) family and functions as a molecular sensor of intracellular heme. We thus hypothesized that antioxidants, albeit lowing ROS production, elicit a pro-tumorigenic effect on BTZ-resistant MCL cells through a BACH1-mediated pathway. To test this possibility, we first confirmed that BACH1 protein levels were dramatically increased in REC-1 and BACH2^KD^ cells upon the combination of BTZ and NAC compared with BTZ single treatment (Fig. 4b); however, *BACH1* mRNA levels were not altered (Fig. 4c), suggesting stabilization of BACH1 proteins. Indeed, BACH1 degradation was delayed in NAC-pretreated cells in the presence of cycloheximide (CHX) (Fig. 4d) with an increased half-life (Fig. 4e), indicating that NAC stabilizes BACH1 in BTZ-resistant MCL cells. Intriguingly, further analysis of one published microarray dataset (GSE2350) [17] revealed higher *BACH1* levels in MCL patients compared with other subtypes of lymphomas (Fig. 4f), implying a potential oncogenic role of BACH1 in MCL. More strikingly, the correlation analysis using another published dataset (GSE21452) [18] displayed a significant positive correlation between *BACH1* and *CCND1* expressions in MCL patients (Fig. 4g), and the latter is a hallmark of MCL caused by a chromosomal translocation of t(11;14)(q13;q32). These findings suggested that the elevated BACH1 proteins are probably associated with the pathogenesis and progression of MCL, a new topic that needs further investigation.

### Dual inhibition of PI3K-AKT signaling and autophagy formation by 3-MA sensitizes MCL cells to BTZ in vitro and in vivo

**Fig. 5:**
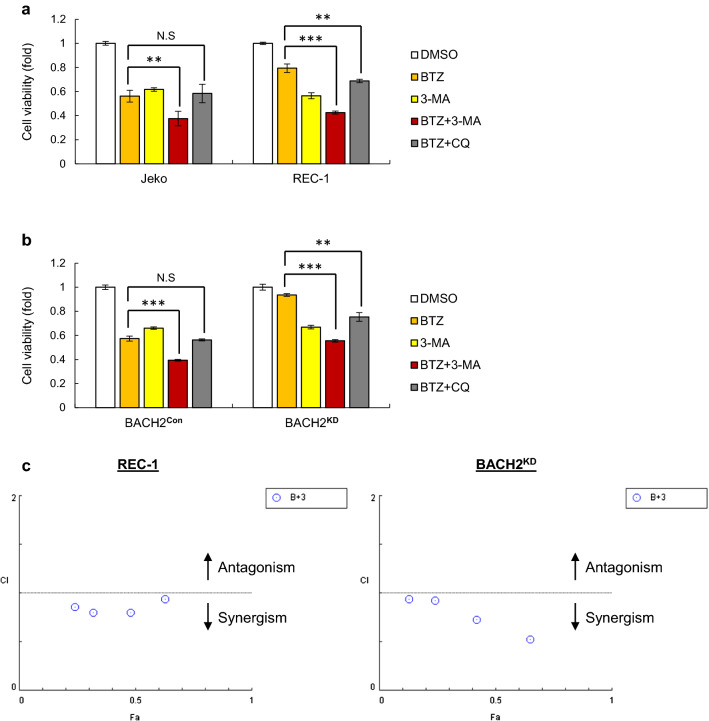
Dual inhibition of PI3K-AKT and autophagy pathways by 3-MA sensitizes MCL cells to BTZ in vitro. Jeko and REC-1 cells (**a**) as well as manipulated Jeko cells (**b**) were treated with BTZ (20 nM) in the presence or absence of 3-MA (5 mM) for 24 h. Cells treated with DMSO, 3-MA or BTZ plus CQ (20 μM) were used as negative controls. Cell viability was measured using MTT assays. Data are normalized to DMSO-treated cells and shown as the mean ± SD from three independent experiments. N.S, not significant; **p < 0.01; ***p < 0.001 (vs BTZ-treated group). **c** The synergic cytotoxic effects of BTZ and 3-MA in REC-1 and BACH2^KD^ Jeko cells were further determined using the combination index (CI) based on the data from cell viability assays. Fa, fraction affected; B, BTZ; 3, 3-MA

Given that antioxidants aggravate BTZ resistance in REC-1 and BACH^KD^ cells, we then attempted to find other agents to overcome chemoresistance. Based on our results, BTZ triggers a strong activation of PI3K signaling and autophagy formation in resistant cells. We thus tried 3-MA, a dual inhibitor of both pathways, as an approach to overcome BTZ resistance. Compared to BTZ single treatment, 3-MA obviously sensitized MCL cells to BTZ, and more significant efficacy of BTZ and 3-MA was observed in resistant REC-1 and BACH2^KD^ Jeko cells (Fig. 5a, b). In contrast, combined treatment with BTZ and chloroquine (CQ), another autophagy inhibitor to block autophagolysosome formation, had no effect on sensitive cells but showed cytotoxic activities on resistant cells compared to BTZ single treatment (Fig. 5a, b), supporting what we found that resistant cells elicit cytoprotective autophagy upon BTZ treatment. However, less efficacy of BTZ and CQ was observed than the combination of BTZ with 3-MA in resistant cells. Further analyses of synergistic cytotoxic effects of BTZ and 3-MA in resistant cells were all less than 1, indicating dramatic synergistic responses (Fig. 5c).

**Fig. 6:**
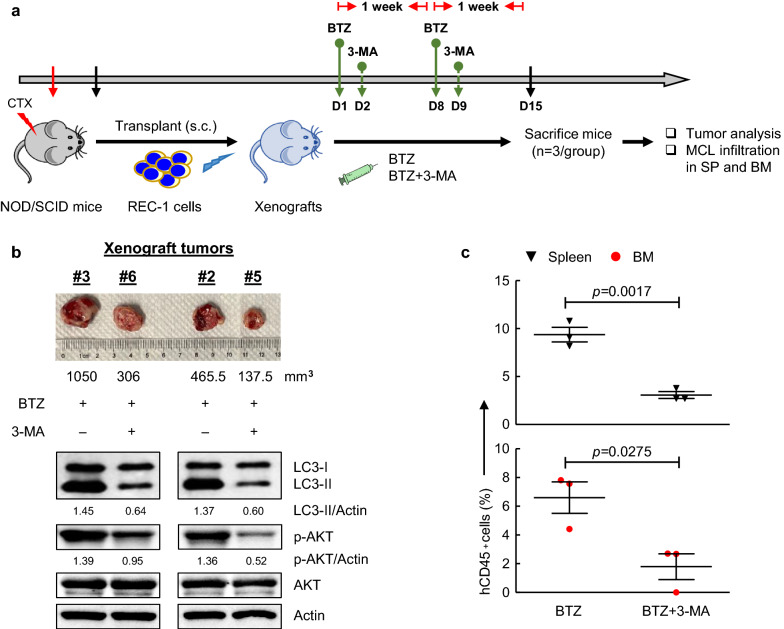
The synergistic effects of BTZ and 3-MA in vivo. **a** Experimental schema for testing the efficacy of BTZ and 3-MA in vivo. Resistant REC-1 cells (5 × 10^6^) were subcutaneously (s.c.) injected into NOD/SCID mice (n = 6). After the average tumor volume reached 50 mm^**3**^, xenografts #1–#3 were treated with BTZ alone (on Day 1 and 8) and xenografts #4–#6 were treated with BTZ (on Day 1 and 8) and 3-MA (on Day 2 and 9) for two weeks. Xenografts were then sacrificed on Day 15 for further analysis. D, day. **b** Representative s.c. tumors were photographed against a centimeter ruler (top), with their volumes under the photo. LC3II, p-AKT and AKT in tumor samples were measured using immunoblotting with Actin as a loading control (bottom). **c** Human CD45 (hCD45^**+**^) cells from spleen (SP) and bone marrow (BM) were analyzed using FCM. Data are presented as the mean ± SEM

To determine whether combined treatment with BTZ and 3-MA is also effective in MCL xenografts, we subcutaneously transplanted resistant REC-1 cells into mice. Tumors were allowed to grow until the average tumor volume reached 50 mm^3^. Xenografts were then treated with BTZ with or without 3-MA (Fig. 6a). Following two-week treatment, the tumor volumes in the xenografts co-treated with BTZ and 3-MA (#5, #6) were about 70% smaller than those treated with BTZ alone (#2, #3) (Fig. 6b). Furthermore, combined treatment with BTZ and 3-MA demonstrated a remarkable decrease in p-AKT levels and autophagy formation in tumor cells (Fig. 6b), and led to a more effective inhibition of MCL infiltration in SP and BM compared with the single-agent group (Fig. 6c). These findings supported an effective combination therapy of BTZ and 3-MA to alleviate the progression of MCL in tumor-bearing xenografts.

## Discussion

Despite the influx of novel agents into the therapeutic strategy during the past decades, there has been no significant advance in the management of patients with MCL. BTZ is the first proteasome inhibitor in clinical use, and resistance to BTZ in MCL patients remains a great challenge. Therefore, a better understanding of the mechanisms underlying the BTZ resistance in MCL is indispensable for developing individualized therapy that is efficient and safe for treating MCL and other hematological malignancies.

ROS has been recognized as a key factor to stimulate tumor initiation and development. As a consequence, cancer patients are willing to supplement their diets with antioxidants such as NAC. However, faced with mounting evidence from bench to clinical trials, scientists have realized that ROS plays a role like a double-edged sword in tumorigenesis and progression. In the early events of tumor initiation, ROS functions as a tumor-promoting factor by inducing genomic instability, oncogene activation and multiple metabolic alterations [30, 31]. Once a tumor forms and develops, tumor cells can express increased levels of antioxidant proteins to detoxify the ROS, creating a delicate balance of intracellular ROS levels that are required for cancer cell survival. On the other hand, chemotherapeutic agents, like BTZ, induce excessive intracellular ROS to reach a toxic level in cancer cells, which, however, can be repulsed by the cancer cells via enhanced antioxidant capacity, leading to treatment failure or drug resistance [30, 31]. The fact that ROS plays a controversial role makes pro-oxidant chemotherapy a very challenging area of study. Indeed, we discovered a cytotoxic role of BTZ-induced excessive ROS in sensitive cells, whereas in BTZ-resistant cells, dysfunction of BACH2 causes high basal and inducible levels of HO-1 to promote antioxidant defense, thereby maintaining ROS in a certain amount that allows pro-tumorigenic signaling without inducing cell death. It is hard to determine the bifurcation point of ROS from tumorigenic to cytotoxic impact on cancer cells, and the relative ROS-adaptative mechanisms in different stages of cancer development remain ambiguous.

To protect cells against toxic ROS, the human body is equipped with an antioxidant system where HO-1 is a key factor. We identified that inhibition of HO-1 by pharmacological approach with SnPP sensitizes MCL cells to BTZ by increasing cytotoxic ROS and reducing cytoprotective autophagy, suggesting multiple functions of HO-1 in MCL. In fact, similar roles of HO-1 have been reported in other blood cancers. For example, upregulated HO-1 is associated with chemoresistance in multiple myeloma (MM) [32] and chronic myeloid leukemia [33]. Inhibition of HO-1 enhances sensitivity to BTZ in MM cells via ERK/STAT3 axis [34]. Nonetheless, it remains unclear whether there are any other non-canonical roles of HO-1 in MCL chemoresistance. In addition, our finding of BACH2-HO-1 signaling adds a new layer in the understanding of how BACH2 blockade contributes to BTZ resistance in MCL.

Although mild increase in BTZ-induced ROS contributes to BTZ resistance in MCL, simply blocking ROS by antioxidant NAC does not cause cell death but facilitates MCL survival upon BTZ treatment. One explanation of the failure in NAC treatment might be due to the fact that it triggers alternative pro-tumorigenic or pro-survival signaling pathways. True as it is, pretreatment of NAC stabilized BACH1 in resistant MCL cells, and this finding is supported by recent studies demonstrating the tumor-promoting signature of stabilized BACH1 in human solid tumors [27–29]. It is of interest to note that BACH1 and BACH2, the only two members of BACH family, play important roles in the regulation of oxidative stress [35]. For example, both of them serve as molecular sensors of heme-driven ROS by controlling the expression of antioxidant genes [24, 36]. With the evolution of adaptive immunity, BACH1 and BACH2 gain essential non-redundant roles in myeloid and lymphoid lineages, respectively [37]. Strikingly, the functions of them in human cancers seem to be the opposite. The nuclear BACH1 expression is associated with adverse clinical features in DLBCL [38], whereas BACH2 has been well-recognized as a tumor suppressor in many hematological malignancies (13, 22, 39–42). However, the biological function of BACH1 in MCL remains unknown. Our analyses revealed higher *BACH1* expression in MCL than other lymphoma subtypes, and a significant positive correlation between *BACH1* and *CCND1* was also discovered in patients with MCL, implying a potential oncogenic role of BACH1. The exact mechanisms of how elevated BACH1 promotes MCL survival and which signaling is involved are worth further exploration.

Given the higher costs and time-consuming issues in developing novel agents, combination of conventional medicine has become a more effective strategy to achieve synergistic anti-tumor effects or overcome chemoresistance. Our finding of enhanced PI3K and autophagy pathways in BTZ-resistant cells allowed us to utilize 3-MA as a sensitizer. As expected, 3-MA remarkably synergized with BTZ to battle against BTZ-resistant cells. Our experiments with MCL xenografts treated with BTZ and 3-MA further provided strong evidence for this.

## Conclusion

**Fig. 7:**
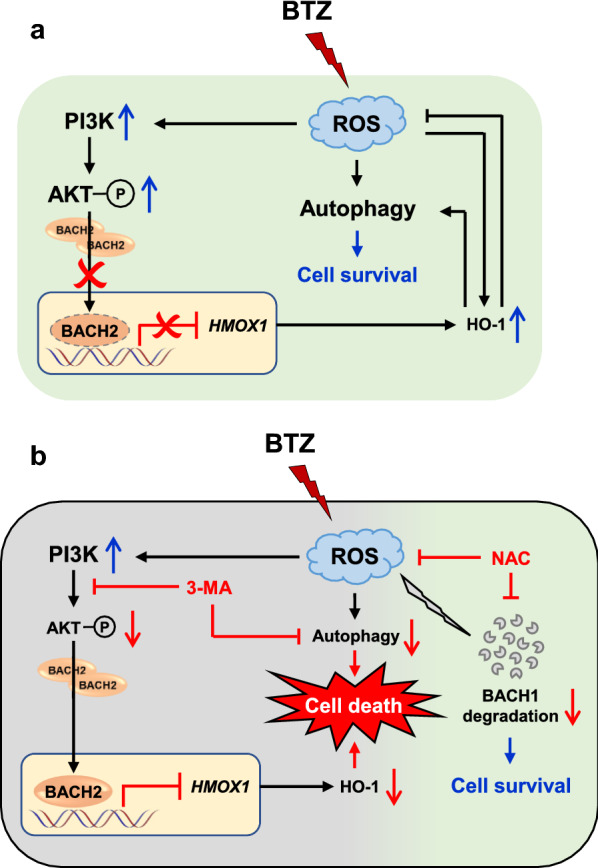
The interconnected molecular events in resistant MCL cells upon BTZ treatment. **a** In BTZ-resistant MCL cells, BTZ-induced ROS enhances PI3K-AKT signaling and cytoprotective autophagy. Activation of PI3K-AKT pathway blocks nuclear translocation of BACH2, thus removing the transcriptional repression of BACH2 on *HMOX1*. Increased HO-1 further maintains ROS in a relative lower tumor-promoting level and induces autophagy formation, leading to cell survival. **b** 3-MA increases BTZ-induced cytotoxicity via dual inhibition of PI3K-AKT pathway and autophagy formation. Particular attention should be paid to the antioxidants supplements which aggravate BTZ resistance by stabilizing BACH1

In summary, ROS-induced PI3K-AKT activation and autophagy induction are involved in the mechanism underlying BTZ resistance in MCL. Enhanced PI3K-AKT pathway augments antioxidant reaction via blocking nuclear translocation of BACH2, which consequently contributes to upregulation of HO-1. Increased HO-1 further keeps ROS within a minimal tumor-promoting level and enhances cytoprotective autophagy (Fig. 7a). In this case, the use of 3-MA sensitizes resistant MCL cells to BTZ treatment through dual inhibition of PI3K-AKT pathway and autophagy formation, thereby increasing cytotoxicity. On the other hand, MCL patients should pay particular attention to the supplement with antioxidants (e.g., NAC), which aggravate BTZ resistance via stabilizing BACH1 (Fig. 7b). Future studies that fully characterize ROS-adaptative mechanisms in MCL, especially in resistant cases, will be required for tailored and individualized treatment in MCL patients.

## Data Availability

The data in the current study are available from the corresponding author on reasonable request.

## Abbreviations

3-MA: 3-Methyladenine
BACH1: BTB and CNC homology 1
BACH2: BTB and CNC homology 2
BL: Burkitt lymphoma
BM: Bone marrow
BTZ: Bortezomib
bZIP: Basic region-leucine zipper
CHX: Cycloheximide
CI: Combination index
CQ: Chloroquine
DLBCL: Diffuse large B cell lymphoma
FCM: Flow cytometry
FDA: Food and Drug Administration
FL: Follicular lymphoma
HMOX1/HO-1: Heme oxygenase-1
MCL: Mantle cell lymphoma
MFI: Mean fluorescence intensity
MM: Multiple myeloma
NAC: *N*-Acetylcysteine
NHL: Non-Hodgkin’s lymphoma
NOD/SCID: Non-obese diabetic/severe combined immunodeficient
p-AKT: Phosphorylated AKT
PEL: Primary effusion lymphoma
ROS: Reactive oxygen species
SEM: Standard error of mean
SD: Standard deviation
SnPP: Tin protoporphyrin IX dichloride
SP: Spleen
